# *N*,*N*′-Diphenyl­suberamide

**DOI:** 10.1107/S1600536810017344

**Published:** 2010-05-15

**Authors:** B. Thimme Gowda, Miroslav Tokarčík, Vinola Z. Rodrigues, Jozef Kožíšek, Hartmut Fuess

**Affiliations:** a Department of Chemistry, Mangalore University, Mangalagangotri 574 199, Mangalore, India; b Faculty of Chemical and Food Technology, Slovak Technical University, Radlinského 9, SK-812 37 Bratislava, Slovak Republic; c Institute of Materials Science, Darmstadt University of Technology, Petersenstrasse 23, D-64287 Darmstadt, Germany

## Abstract

In the title compound (systematic name: *N*,*N*′-diphenyl­octanediamide), C_20_H_24_N_2_O_2_, the two phenyl rings make an inter­planar angle of 76.5 (2)°. The crystal structure is stabilized by inter­molecular N—H⋯O hydrogen bonds, which link the mol­ecules into chains running along the *b* axis. The crystal studied was non-merohedrally twinned, the fractional contribution of the minor twin component being 0.203 (2).

## Related literature

For related structures, see: Gowda *et al.* (2007 ▶, 2009**a* ▶,b* ▶).
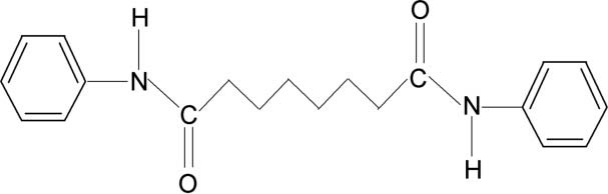


## Experimental

### 

#### Crystal data

C_20_H_24_N_2_O_2_
*M* *_r_* = 324.41
Monoclinic, 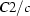

*a* = 18.2267 (9) Å
*b* = 5.03097 (15) Å
*c* = 38.1436 (15) Å
β = 96.517 (4)°
*V* = 3475.1 (2) Å^3^
*Z* = 8
Mo *K*α radiation
μ = 0.08 mm^−1^
*T* = 295 K
0.58 × 0.33 × 0.05 mm

#### Data collection

Oxford Diffraction Gemini R CCD diffractometer
Absorption correction: analytical (*CrysAlis PRO*; Oxford Diffraction, 2009 ▶) *T*_min_ = 0.957, *T*_max_ = 0.996
27788 measured reflections
3027 independent reflections
2524 reflections with *I* > 2σ(*I*)
*R*_int_ = 0.064

#### Refinement

*R*[*F* ^2^ > 2σ(*F* ^2^)] = 0.076
*wR*(*F* ^2^) = 0.203
*S* = 1.09
3027 reflections
224 parameters
2 restraints
H atoms treated by a mixture of independent and constrained refinement
Δρ_max_ = 0.21 e Å^−3^
Δρ_min_ = −0.22 e Å^−3^

### 

Data collection: *CrysAlis PRO* (Oxford Diffraction, 2009 ▶); cell refinement: *CrysAlis PRO*; data reduction: *CrysAlis PRO*; program(s) used to solve structure: *SHELXS97* (Sheldrick, 2008 ▶); program(s) used to refine structure: *SHELXL97* (Sheldrick, 2008 ▶); molecular graphics: *ORTEP-3* (Farrugia, 1997 ▶) and *DIAMOND* (Brandenburg, 2002 ▶); software used to prepare material for publication: *SHELXL97*, *PLATON* (Spek, 2009 ▶) and *WinGX* (Farrugia, 1999 ▶).

## Supplementary Material

Crystal structure: contains datablocks I, global. DOI: 10.1107/S1600536810017344/bt5267sup1.cif

Structure factors: contains datablocks I. DOI: 10.1107/S1600536810017344/bt5267Isup2.hkl

Additional supplementary materials:  crystallographic information; 3D view; checkCIF report

## Figures and Tables

**Table 1:** Hydrogen-bond geometry (Å, °)

*D*—H⋯*A*	*D*—H	H⋯*A*	*D*⋯*A*	*D*—H⋯*A*
N1—H1*N*⋯O1^i^	0.84 (3)	2.17 (3)	3.004 (4)	173 (4)
N2—H2*N*⋯O2^ii^	0.84 (3)	2.13 (3)	2.937 (4)	161 (4)

Symmetry codes: (i) 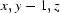; (ii) 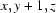.

**Fig. 1.**
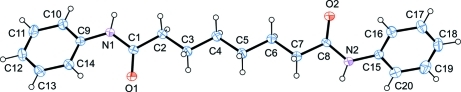
Molecular structure of (I) showing the atom labelling scheme. Displacement ellipsoids are drawn at the 30% probability level and H atoms are represented as small spheres of arbitrary radii.

**Fig. 2.**
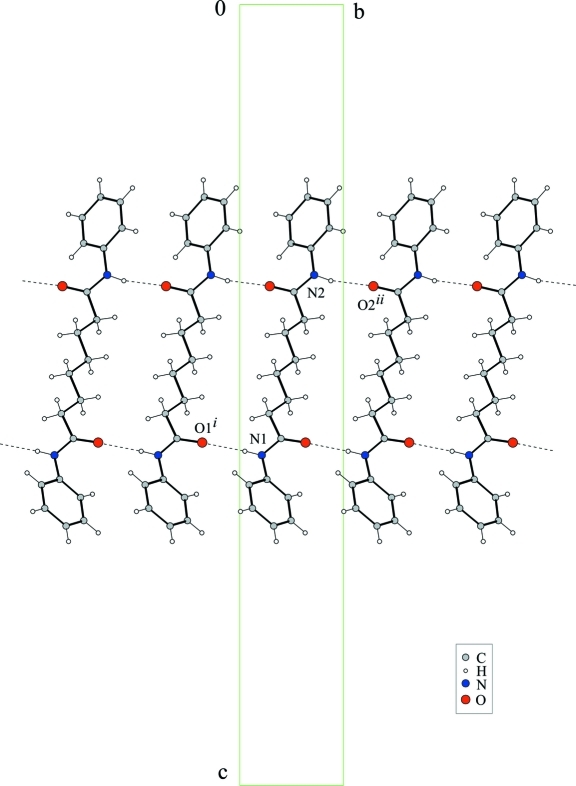
Part of crystal structure of (I) viewed down the a-axis. Intermolecular N–H···O hydrogen bonds (shown as dashed lines) connect the molecules into chains running along the b-axis of the crystal. Symmetry codes (i): x, y-1, z; (ii): x, y+1, z.

## supplementary crystallographic information

### Comment

The amide moiety is an important constituent of many biologically significant compounds. As a part of studying the effect of ring and side chain substitutions on the structures of this class of compounds (Gowda *et al.*, 2007; 2009*a,b*), the crystal structure of *N*,*N*-bis(phenyl)-suberamide has been determined (I) (Fig. 1).

In the structure, the two phenyl rings make an interplanar angle of 76.5 (2)°. The plane of the aliphatic group C2/C7 makes dihedral angles of 26.3 (5)° with the amide group (N1, H1N, C1, O1) and 27.2 (5)° with the amide group (N2, H2N, C8, O2). The conformations of the amide groups with respect to the attached phenyl rings are given by the torsion angles of C14—C9—N1—C1 = -38.0 (6)° and C16—C15—N2—C8 = -42.2 (6)°. The structure is stabilized by two intramolecular hydrogen bonds (Table 1). The intermolecular N–H···O hydrogen bonds link the molecules into the chains running along the *b*-axis of the crystal (Fig. 2). The crystal is merohedrally twinned with the twin fraction of 0.203 (2).

### Experimental

Suberic acid (0.3 mol) was heated with thionyl chloride (1.2 mol) at 120°C for 4 hours. The acid chloride obtained was treated with aniline (0.6 mol). The product obtained was added to crushed ice to obtain the white precipitate. It was thoroughly washed with water and then with saturated sodium bicarbonate solution and washed again with water. It was then given a wash with 2 N HCl. It was again washed with water, filtered, dried and recrystallised to constant point (186-188°C) from ethanol-Tetrahydrofuran mixture in the ratio 1:4.

Plate like colourless single crystals of the title compound used in X-ray diffraction studies were obtained by a slow evaporation of its solution at room temperature.

### Refinement

The crystal used for data collection was a non-merohedral twin. The twin law was found to be a twofold axis about the [1 0 4] direct lattice direction. The final refinement was made using the HKLF4 format of the HKL file, and using the INS file having the twin matrix (-1 0 0 / 0 -1 0 / 0.5 0 1) in the TWIN instruction. The fractional contribution of the minor twin component refined to 0.203 (2). The C-bounded hydrogen atoms were positioned with idealized geometry using a riding model with C–H = 0.93 Å or 0.97 Å. Amide H atoms were refined with N–H distance restrained to 0.85 (3) Å. The *U*_iso_(H) values were set at 1.2*U*_eq_(C, N).

### Crystal data

**Table:**

C_20_H_24_N_2_O_2_	*F*(000) = 1392
*M**_r_* = 324.41	*D*_x_ = 1.24 Mg m^−^^3^
Monoclinic, *C*2/*c*	Mo *K*α radiation, λ = 0.71073 Å
Hall symbol: -C 2yc	Cell parameters from 7864 reflections
*a* = 18.2267 (9) Å	θ = 1.9–27.4°
*b* = 5.03097 (15) Å	µ = 0.08 mm^−^^1^
*c* = 38.1436 (15) Å	*T* = 295 K
β = 96.517 (4)°	Plate, colourless
*V* = 3475.1 (2) Å^3^	0.58 × 0.33 × 0.05 mm
*Z* = 8	

### Data collection

**Table:**

Oxford Diffraction Gemini R CCD diffractometer	3027 independent reflections
graphite	2524 reflections with *I* > 2σ(*I*)
Detector resolution: 10.434 pixels mm^-1^	*R*_int_ = 0.064
ω scans	θ_max_ = 25.0°, θ_min_ = 2.2°
Absorption correction: analytical (*CrysAlis PRO*; Oxford Diffraction, 2009)	*h* = −21→21
*T*_min_ = 0.957, *T*_max_ = 0.996	*k* = −5→5
27788 measured reflections	*l* = −45→45

### Refinement

**Table:**

Refinement on *F*^2^	Primary atom site location: structure-invariant direct methods
Least-squares matrix: full	Secondary atom site location: difference Fourier map
*R*[*F*^2^ > 2σ(*F*^2^)] = 0.076	Hydrogen site location: inferred from neighbouring sites
*wR*(*F*^2^) = 0.203	H atoms treated by a mixture of independent and constrained refinement
*S* = 1.09	*w* = 1/[σ^2^(*F*_o_^2^) + (0.0865*P*)^2^ + 8.373*P*] where *P* = (*F*_o_^2^ + 2*F*_c_^2^)/3
3027 reflections	(Δ/σ)_max_ < 0.001
224 parameters	Δρ_max_ = 0.21 e Å^−^^3^
2 restraints	Δρ_min_ = −0.22 e Å^−^^3^

### Special details

**Table:**

Geometry. All esds (except the esd in the dihedral angle between two l.s. planes) are estimated using the full covariance matrix. The cell esds are taken into account individually in the estimation of esds in distances, angles and torsion angles; correlations between esds in cell parameters are only used when they are defined by crystal symmetry. An approximate (isotropic) treatment of cell esds is used for estimating esds involving l.s. planes.
Refinement. Refinement of *F*^2^ against ALL reflections. The weighted *R*-factor wR and goodness of fit *S* are based on *F*^2^, conventional *R*-factors *R* are based on *F*, with *F* set to zero for negative *F*^2^. The threshold expression of *F*^2^ > σ(*F*^2^) is used only for calculating *R*-factors(gt) etc. and is not relevant to the choice of reflections for refinement. *R*-factors based on *F*^2^ are statistically about twice as large as those based on *F*, and *R*- factors based on ALL data will be even larger.

### Fractional atomic coordinates and isotropic or equivalent isotropic displacement parameters (Å^2^)

**Table:**

	*x*	*y*	*z*	*U*_iso_*/*U*_eq_	
C1	0.0479 (2)	0.3940 (7)	0.55627 (10)	0.0357 (9)	
C2	0.0772 (2)	0.2805 (8)	0.52403 (10)	0.0392 (9)	
H2A	0.0358	0.2378	0.5067	0.047*	
H2B	0.1029	0.1158	0.5306	0.047*	
C3	0.1291 (2)	0.4621 (8)	0.50714 (10)	0.0398 (9)	
H3A	0.1732	0.489	0.5235	0.048*	
H3B	0.1055	0.6337	0.5028	0.048*	
C4	0.1511 (2)	0.3560 (8)	0.47276 (10)	0.0398 (9)	
H4A	0.1068	0.3257	0.4567	0.048*	
H4B	0.1754	0.1857	0.4772	0.048*	
C5	0.2020 (2)	0.5373 (8)	0.45507 (9)	0.0415 (10)	
H5A	0.247	0.5627	0.4709	0.05*	
H5B	0.1784	0.7095	0.4514	0.05*	
C6	0.2222 (2)	0.4363 (8)	0.42003 (10)	0.0417 (10)	
H6A	0.2457	0.2638	0.4235	0.05*	
H6B	0.1775	0.4133	0.404	0.05*	
C7	0.2735 (2)	0.6215 (8)	0.40346 (10)	0.0416 (10)	
H7A	0.3186	0.6418	0.4194	0.05*	
H7B	0.2505	0.795	0.4004	0.05*	
C8	0.2930 (2)	0.5254 (7)	0.36811 (10)	0.0388 (9)	
C9	−0.0140 (2)	0.2611 (7)	0.60821 (9)	0.0343 (8)	
C10	−0.0691 (2)	0.0877 (9)	0.61530 (10)	0.0459 (10)	
H10	−0.0822	−0.0522	0.5999	0.055*	
C11	−0.1051 (3)	0.1199 (10)	0.64514 (11)	0.0565 (12)	
H11	−0.1425	0.0034	0.6497	0.068*	
C12	−0.0851 (3)	0.3247 (10)	0.66783 (11)	0.0595 (13)	
H12	−0.109	0.3482	0.6879	0.071*	
C13	−0.0299 (3)	0.4945 (9)	0.66109 (10)	0.0574 (12)	
H13	−0.0168	0.633	0.6767	0.069*	
C14	0.0070 (2)	0.4649 (8)	0.63144 (10)	0.0439 (10)	
H14	0.0451	0.5798	0.6273	0.053*	
C15	0.3338 (2)	0.6863 (7)	0.31213 (10)	0.0382 (9)	
C16	0.3837 (2)	0.4959 (9)	0.30577 (11)	0.0517 (11)	
H16	0.401	0.3772	0.3235	0.062*	
C17	0.4088 (3)	0.4791 (11)	0.27288 (13)	0.0676 (14)	
H17	0.4425	0.3479	0.2685	0.081*	
C18	0.3837 (3)	0.6570 (11)	0.24667 (12)	0.0695 (15)	
H18	0.4011	0.6476	0.2247	0.083*	
C19	0.3336 (3)	0.8462 (11)	0.25299 (13)	0.0738 (16)	
H19	0.3157	0.9633	0.2352	0.089*	
C20	0.3091 (3)	0.8644 (9)	0.28602 (12)	0.0565 (12)	
H20	0.2759	0.9971	0.2905	0.068*	
N1	0.02190 (19)	0.2112 (6)	0.57763 (8)	0.0381 (8)	
H1N	0.029 (2)	0.055 (6)	0.5713 (11)	0.046*	
N2	0.3100 (2)	0.7191 (6)	0.34624 (9)	0.0405 (8)	
H2N	0.302 (2)	0.868 (6)	0.3547 (11)	0.049*	
O1	0.04456 (18)	0.6348 (5)	0.56139 (8)	0.0503 (8)	
O2	0.2948 (2)	0.2890 (6)	0.36057 (9)	0.0652 (10)	

### Atomic displacement parameters (Å^2^)

**Table:**

	*U*^11^	*U*^22^	*U*^33^	*U*^12^	*U*^13^	*U*^23^
C1	0.041 (2)	0.029 (2)	0.038 (2)	0.0025 (17)	0.0096 (17)	−0.0019 (17)
C2	0.050 (2)	0.032 (2)	0.038 (2)	−0.0012 (18)	0.0144 (18)	−0.0054 (17)
C3	0.046 (2)	0.037 (2)	0.038 (2)	−0.0056 (18)	0.0141 (17)	−0.0075 (17)
C4	0.044 (2)	0.038 (2)	0.039 (2)	−0.0018 (18)	0.0125 (17)	−0.0048 (17)
C5	0.052 (2)	0.038 (2)	0.037 (2)	−0.0057 (19)	0.0163 (18)	−0.0080 (18)
C6	0.055 (2)	0.031 (2)	0.042 (2)	−0.0081 (18)	0.0166 (19)	−0.0062 (17)
C7	0.051 (2)	0.033 (2)	0.042 (2)	−0.0046 (19)	0.0131 (19)	−0.0038 (18)
C8	0.050 (2)	0.026 (2)	0.044 (2)	−0.0011 (18)	0.0215 (19)	−0.0072 (17)
C9	0.039 (2)	0.0295 (19)	0.0347 (19)	0.0036 (17)	0.0069 (16)	0.0021 (15)
C10	0.057 (3)	0.043 (2)	0.039 (2)	−0.007 (2)	0.0131 (19)	−0.0037 (18)
C11	0.060 (3)	0.058 (3)	0.056 (3)	0.002 (2)	0.029 (2)	0.011 (2)
C12	0.079 (3)	0.062 (3)	0.042 (2)	0.013 (3)	0.030 (2)	0.005 (2)
C13	0.089 (3)	0.047 (3)	0.037 (2)	0.005 (3)	0.011 (2)	−0.009 (2)
C14	0.051 (2)	0.040 (2)	0.041 (2)	−0.0034 (19)	0.0109 (19)	−0.0051 (19)
C15	0.046 (2)	0.030 (2)	0.041 (2)	−0.0092 (17)	0.0134 (18)	−0.0034 (17)
C16	0.060 (3)	0.049 (2)	0.050 (2)	0.008 (2)	0.021 (2)	0.002 (2)
C17	0.081 (3)	0.060 (3)	0.070 (3)	0.005 (3)	0.042 (3)	−0.009 (3)
C18	0.102 (4)	0.066 (3)	0.046 (3)	−0.018 (3)	0.035 (3)	−0.009 (3)
C19	0.113 (5)	0.063 (3)	0.047 (3)	−0.005 (3)	0.019 (3)	0.013 (3)
C20	0.072 (3)	0.042 (3)	0.058 (3)	0.002 (2)	0.017 (2)	0.004 (2)
N1	0.0527 (19)	0.0262 (16)	0.0384 (17)	−0.0009 (15)	0.0174 (15)	−0.0018 (14)
N2	0.054 (2)	0.0263 (17)	0.0446 (19)	−0.0037 (15)	0.0198 (16)	−0.0016 (15)
O1	0.072 (2)	0.0293 (15)	0.0548 (18)	0.0002 (14)	0.0316 (16)	−0.0006 (13)
O2	0.110 (3)	0.0280 (16)	0.067 (2)	−0.0056 (17)	0.051 (2)	−0.0043 (14)

### Geometric parameters (Å, °)

**Table:**

C1—O1	1.229 (5)	C9—N1	1.423 (5)
C1—N1	1.350 (5)	C10—C11	1.386 (6)
C1—C2	1.508 (5)	C10—H10	0.93
C2—C3	1.510 (5)	C11—C12	1.368 (7)
C2—H2A	0.97	C11—H11	0.93
C2—H2B	0.97	C12—C13	1.366 (7)
C3—C4	1.512 (5)	C12—H12	0.93
C3—H3A	0.97	C13—C14	1.388 (6)
C3—H3B	0.97	C13—H13	0.93
C4—C5	1.513 (5)	C14—H14	0.93
C4—H4A	0.97	C15—C16	1.362 (6)
C4—H4B	0.97	C15—C20	1.377 (6)
C5—C6	1.514 (5)	C15—N2	1.427 (5)
C5—H5A	0.97	C16—C17	1.385 (6)
C5—H5B	0.97	C16—H16	0.93
C6—C7	1.509 (5)	C17—C18	1.381 (7)
C6—H6A	0.97	C17—H17	0.93
C6—H6B	0.97	C18—C19	1.360 (8)
C7—C8	1.512 (5)	C18—H18	0.93
C7—H7A	0.97	C19—C20	1.387 (7)
C7—H7B	0.97	C19—H19	0.93
C8—O2	1.225 (5)	C20—H20	0.93
C8—N2	1.342 (5)	N1—H1N	0.84 (3)
C9—C10	1.380 (6)	N2—H2N	0.84 (3)
C9—C14	1.381 (5)		
			
O1—C1—N1	123.3 (3)	C10—C9—C14	119.9 (3)
O1—C1—C2	122.1 (3)	C10—C9—N1	117.5 (3)
N1—C1—C2	114.5 (3)	C14—C9—N1	122.5 (3)
C1—C2—C3	114.6 (3)	C9—C10—C11	120.7 (4)
C1—C2—H2A	108.6	C9—C10—H10	119.7
C3—C2—H2A	108.6	C11—C10—H10	119.7
C1—C2—H2B	108.6	C12—C11—C10	119.3 (4)
C3—C2—H2B	108.6	C12—C11—H11	120.3
H2A—C2—H2B	107.6	C10—C11—H11	120.3
C2—C3—C4	113.4 (3)	C13—C12—C11	120.1 (4)
C2—C3—H3A	108.9	C13—C12—H12	119.9
C4—C3—H3A	108.9	C11—C12—H12	119.9
C2—C3—H3B	108.9	C12—C13—C14	121.4 (4)
C4—C3—H3B	108.9	C12—C13—H13	119.3
H3A—C3—H3B	107.7	C14—C13—H13	119.3
C3—C4—C5	114.2 (3)	C9—C14—C13	118.5 (4)
C3—C4—H4A	108.7	C9—C14—H14	120.7
C5—C4—H4A	108.7	C13—C14—H14	120.7
C3—C4—H4B	108.7	C16—C15—C20	120.0 (4)
C5—C4—H4B	108.7	C16—C15—N2	121.5 (4)
H4A—C4—H4B	107.6	C20—C15—N2	118.4 (4)
C4—C5—C6	114.5 (3)	C15—C16—C17	120.0 (4)
C4—C5—H5A	108.6	C15—C16—H16	120
C6—C5—H5A	108.6	C17—C16—H16	120
C4—C5—H5B	108.6	C18—C17—C16	120.0 (5)
C6—C5—H5B	108.6	C18—C17—H17	120
H5A—C5—H5B	107.6	C16—C17—H17	120
C7—C6—C5	112.8 (3)	C19—C18—C17	119.9 (4)
C7—C6—H6A	109	C19—C18—H18	120
C5—C6—H6A	109	C17—C18—H18	120
C7—C6—H6B	109	C18—C19—C20	120.0 (5)
C5—C6—H6B	109	C18—C19—H19	120
H6A—C6—H6B	107.8	C20—C19—H19	120
C6—C7—C8	113.3 (3)	C15—C20—C19	120.1 (5)
C6—C7—H7A	108.9	C15—C20—H20	119.9
C8—C7—H7A	108.9	C19—C20—H20	119.9
C6—C7—H7B	108.9	C1—N1—C9	126.9 (3)
C8—C7—H7B	108.9	C1—N1—H1N	113 (3)
H7A—C7—H7B	107.7	C9—N1—H1N	120 (3)
O2—C8—N2	123.0 (4)	C8—N2—C15	126.8 (3)
O2—C8—C7	122.3 (4)	C8—N2—H2N	110 (3)
N2—C8—C7	114.6 (3)	C15—N2—H2N	123 (3)
			
O1—C1—C2—C3	23.9 (6)	C20—C15—C16—C17	−0.9 (7)
N1—C1—C2—C3	−159.8 (3)	N2—C15—C16—C17	−176.4 (4)
C1—C2—C3—C4	−173.8 (3)	C15—C16—C17—C18	0.7 (8)
C2—C3—C4—C5	179.0 (4)	C16—C17—C18—C19	−1.0 (8)
C3—C4—C5—C6	−178.1 (4)	C17—C18—C19—C20	1.7 (8)
C4—C5—C6—C7	−179.4 (4)	C16—C15—C20—C19	1.5 (7)
C5—C6—C7—C8	−178.9 (4)	N2—C15—C20—C19	177.1 (4)
C6—C7—C8—O2	−29.1 (6)	C18—C19—C20—C15	−1.9 (8)
C6—C7—C8—N2	152.7 (4)	O1—C1—N1—C9	1.5 (7)
C14—C9—C10—C11	1.7 (6)	C2—C1—N1—C9	−174.8 (4)
N1—C9—C10—C11	178.2 (4)	C10—C9—N1—C1	145.5 (4)
C9—C10—C11—C12	−0.6 (7)	C14—C9—N1—C1	−38.0 (6)
C10—C11—C12—C13	−0.2 (7)	O2—C8—N2—C15	−1.4 (7)
C11—C12—C13—C14	0.0 (7)	C7—C8—N2—C15	176.8 (4)
C10—C9—C14—C13	−2.0 (6)	C16—C15—N2—C8	−42.2 (6)
N1—C9—C14—C13	−178.3 (4)	C20—C15—N2—C8	142.3 (4)
C12—C13—C14—C9	1.1 (7)		

### Hydrogen-bond geometry (Å, °)

**Table:**

*D*—H···*A*	*D*—H	H···*A*	*D*···*A*	*D*—H···*A*
N1—H1N···O1^i^	0.84 (3)	2.17 (3)	3.004 (4)	173 (4)
N2—H2N···O2^ii^	0.84 (3)	2.13 (3)	2.937 (4)	161 (4)

Symmetry codes: (i) *x*, *y*−1, *z*; (ii) *x*, *y*+1, *z*.
